# Effect of Tai Chi on knee pain and muscle strength in middle-aged and older adults with knee osteoarthritis: a randomized controlled trial protocol

**DOI:** 10.1186/s12906-023-04070-0

**Published:** 2023-07-20

**Authors:** Hongyu Yue, Yang Li, Jianwen Ma, Chaoqun Xie, Fangfang Xie, Junhao Cai, Min Fang, Fei Yao

**Affiliations:** 1 Shanghai Municipal Hospital of Traditional Chinese Medicine, Shanghai University of Traditional Chinese Medicine, Shanghai, China; 2 School of Acupuncture-Moxibustion and Tuina, Shanghai University of Traditional Chinese Medicine, Shanghai, China; 3 Shuguang Hospital, Shanghai University of Traditional Chinese Medicine, Shanghai, China

**Keywords:** Tai Chi, Pain, Muscle strength, Knee osteoarthritis, Randomized controlled trial

## Abstract

**Background:**

Knee osteoarthritis (KOA) is a common public health problem and a leading cause of long-term pain, decreased muscle strength, and even disability. Tai Chi has been proved effective and highly recommended for KOA management worldwide. However, little is known about its benefits on quadriceps strength which is closely associated with relieving knee pain. This trial is designed to evaluate the efficacy and safety of Tai Chi on knee pain and muscle strength in middle-aged and older adults with KOA.

**Methods:**

A total of 100 participants will be randomly divided into a Tai Chi group (TC group) (1x/week for 12 weeks) and a control group with a health education and stretching program (1x/week for 12 weeks) with a follow-up period of 6 weeks. The primary outcome is the change of Western Ontario and McMaster Universities (WOMAC) pain subscale at week 12 compared with baseline. Secondary outcomes include WOMAC stiffness and function subscales, data from isokinetic dynamometry, gait analysis with electromyography (EMG), and a 36-item short form health survey (SF-36). The daily dose of pain-relieving medication will also be recorded. All adverse effects will be assessed by the Treatment Emergent Symptom Scale (TESS).

**Discussion:**

We expect this randomized trial to evaluate the effectiveness of Tai Chi on relieving pain and increasing quadriceps strength in KOA patients. This protocol, if proven effective, will contribute to providing a promising alternative intervention for middle-aged and older adults with KOA.

**Trials registration number:**

This trial has been registered in the China Clinical Trials Registry (registration number: ChiCTR2300069339).

## Introduction

Knee osteoarthritis (KOA) is a common degenerative joint disease with symptoms of long-term pain, disability and reduced quality of life, resulting in heavy personal and social burdens for middle-aged and older people. With the aging population accelerating, KOA has already become one of the most common public health problems in the world. The overall incidence and prevalence of knee OA was reported to be 10 times higher in the 30 to 65 age group than in younger age group, affecting nearly 33.6% of people over 65 [1]. In China, the prevalence rate of KOA for people over 55 was 13.2% in 2017, which has also been increasing annually, and the population tends to be diagnosed at a younger age [2]. These results lead us to find appropriate therapies that are more effective and easier to treat for participating patients.

As reported [3, 4], pain appears to be the most common symptom of KOA, and increasing pain intensity is closely associated with worsening physical functioning in KOA patients. Due to pain, many KOA patients become afraid of stretching and flexing joints, gradually leading to a decreased quality of life and worsening muscle weakness [5, 6]. The most obvious muscle weakness is the decrease in extension and flexion strength in KOA patients, especially quadriceps weakness, which may lead to an increased risk of falls, functional limitation, and disability [7, 8]. Several studies [9, 10] have found that increasing the muscle strength of the quadriceps can effectively relieve knee pain. Moreover, increasing muscle strength has also been shown to be beneficial in alleviating reduced joint mobility and slowing down KOA degeneration effectively [11, 12]. Therefore, improving muscle weakness, especially in the quadriceps, is important for KOA treatment.

Currently available treatments for KOA, including medical therapy, exercise therapy, and surgical therapy, vary from state of disease progression, which are expected to relieve pain, improve the range of motion and function of knee joints, slow down KOA degeneration, and improve patients’ quality of life [13]. Medical treatments such as nonsteroidal anti-inflammatory drugs fail to control pain, relieve other symptoms, and even lead to severe side effects, such as bleeding from the upper gastrointestinal tract, cardiovascular disease, and worsening liver and kidney function [14–16]. Surgery cannot be performed until all conservative methods have been tried without success [17]. Excepting pharmacotherapy and surgery, exercise training may be one way to improve KOA symptoms depending on the cause. Physical exercise has been globally recommended as an effective method of treating KOA by several clinical guidelines [18–20], which are recognized and accepted by the international community. As clinical trials have revealed [21, 22], it is noted that physical exercise has provided limited benefits for pain and physical function of knee joints, but there has been no consensus on the appropriate frequency, intensity and duration of physical exercise to achieve the best curative effect on KOA. Therefore, priority should be given to identifying a novel and effective method of non-pharmaceutical therapies to achieve adequate pain control and improved knee joint function.

As a traditional Chinese mind-body aerobic exercise, TC combines sustained mindfulness, deep diaphragmatic breathing and slow, gentle movements to improve both physical and psychological health. TC has gained worldwide popularity and is widely used in clinical practice, such as falls, Parkinson’s disease, depression, cognitive impairment and dementia, stroke rehabilitation, cardiac disease, and chronic obstructive pulmonary disease [23]. TC has been strongly recommended for KOA management by the American College of Rheumatology as an effective and safe complementary and alternative approach to KOA management to regulate mind and body [24]. Previous studies have indicated that the TC group showed more significant improvements in pain, muscle strength, physical function, and other arthritic symptoms [25–29]. However, little is known about Tai Chi’s benefits on the muscle strength, thus relieving knee pain and improving other KOA symptoms.

We conducted this randomized controlled trial to hypothesize that the application of Tai Chi will lower pain levels, increase quadriceps strength, and improve physical function in KOA patients. The objectives of this trial are (1) to verify that TC provides a positive effect on pain and physical function of KOA in middle-aged and older adults compared to health education, and (2) to explore detained direct correlation between pain and quadriceps strength sufficiently.

## Methods/design

### Study design

**Fig. 1:**
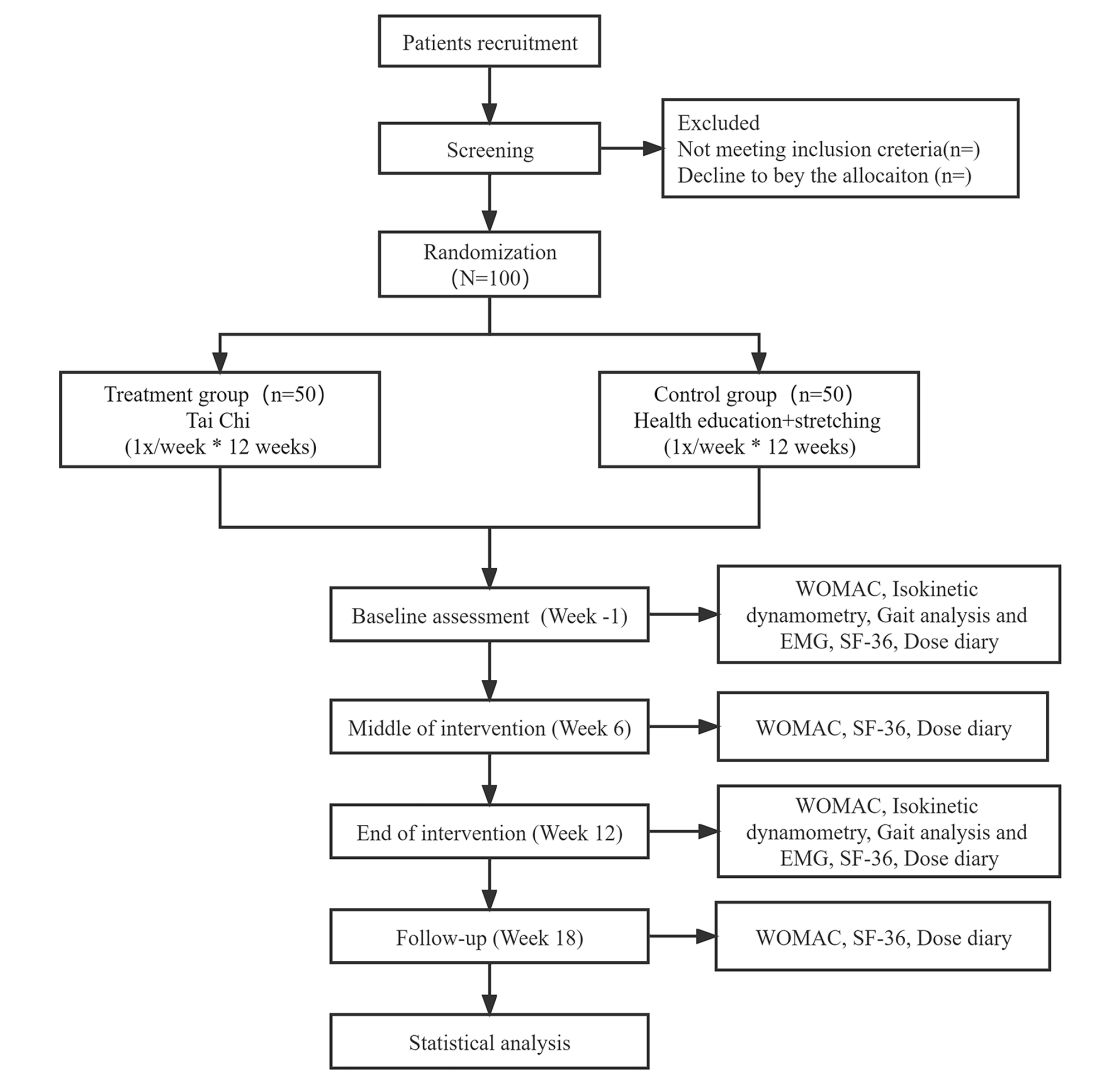
Trial flow chart

This is a single-center, evaluator-blind, randomized controlled trial conducted at Shanghai Municipal Hospital of Traditional Chinese Medicine in China. All eligible participants will be randomly divided at a ratio of 1:1 into Tai Chi group (TC group) (1x/week for 12 weeks) and attention control group (AC group) with health education and stretching program (1x/week for 12 weeks) with a follow-up period of 6 weeks. The primary outcome is the change of Western Ontario and McMaster Universities (WOMAC) pain subscale at week 12 compared with baseline. Secondary outcomes include WOMAC pain subscales at other time-points, stiffness and function subscales, data from isokinetic dynamometry, gait analysis with electromyography (EMG), and a 36-item short form health survey (SF-36). The daily dose of pain-relieving medication will also be recorded. All outcomes will be assessed at baseline (week − 1) and at the end of the intervention (week 12). In addition, WOMAC and SF-36 will also be assessed at 6^th^ week of the intervention (week 6) and at the end of follow-up (week 18). The design of this clinical trial will strictly follow the Consolidated Standards of Reporting Trials (CONSORT) Statement and Standard Protocol Items: Recommendations for Interventional Trials (SPIRIT) [30]. The trial flowchart is presented in Fig. 1, and the study design schedule is shown in Table 1; Fig. 1.

**Table 1:** Trial process chart

	Baseline	Intervention			Follow-up
	Week − 1	Week 0	Week 6	Week 12	Week 18
Patients					
Enrollment	x				
Signed informed consent	x				
Randomization and allocation	x				
Intervention		x	x	x	
Outcomes					
WOMAC-pain	x		x	x	x
WOMAC-stiffness	x		x	x	x
WOAMC-phsical function	x		x	x	x
Isokinetic dynamometry	x			x	
Gait analysis with EMG	x			x	
SF-36	x		x	x	x
Dose of pain-reliving medicine	x	x	x	x	x
Adverse events		x	x	x	x

### Sample size calculation

The calculation of sample size is based on hypothetical changes in WOMAC pain subscale at week 12. We are using the results of a randomized controlled trial [26] to formulate expected effects. We hypothesized that a 118-point improvement in WOMAC pain subscale occurred in the Tai Chi group compared to the attention control group. A sample size of 40 per group provides 90% power to detect these differences using a 2-sided significance level of 0.05 through PASS software (version 15.0.5) and considering the expected dropout rate of 20%, the study will require 100 patients in total.

### Participants recruitment

A total of 100 KOA patients will be recruited from the outpatient departments of Shanghai Municipal Hospital of Traditional Chinese Medicine. Participants will be recruited through online advertisements on official accounts, posters on bulletin boards and flyers at the hospital and local senior centers. Eligible participants will be informed of detailed trial information such as trial benefits and potential adverse reactions, following initial screening criteria for inclusion and exclusion. Interventions and baseline assessments should not be scheduled until written informed consent has been signed.

### Inclusion criteria

1. Meet the diagnostic criteria of KOA set by the American College of Rheumatology [24];
2. Male or female patients aged 40 years or older;
3. Mild to moderate radiographic medial tibiofemoral osteoarthritis (Kellgren - Lawrence score from 1 to 3) in at least one knee;
4. WOMAC pain score ≥ 40 (visual analog version, range 0 to 100, higher indicating more pain) on at least one of five questions;
5. Volunteer to participate in the trial and provide written informed consent.

### Exclusion criteria

1. History of knee surgery or combined with tumor, tuberculosis, osteomyelitis in knee joints;
2. Arthroscopy in the past 6 months or intraarticular injection of steroids or hyaluronic acid in the past 3 months;
3. Major medical or physical conditions determined by doctors to prevent exercise, including serious acute or chronic organic diseases of cardiovascular, hepatic, renal, cerebrovascular and hematopoietic systems, recent stroke, psychiatric disease, active cancer;
4. Have participated in strength training for more than 30 min per week in the past 3 months;
5. Have participated in other clinical trials in the past 4 weeks.

### Randomization and allocation concealment

Recruited participants will begin intervention within 1 week of baseline assessment. An independent statistician will use SPSS (version 26.0) to generate the random sequence with a random block size of 2–6. Eligible participants will be randomly assigned to either the TC group or the control group on a 1:1 ratio. Random numbers and assigned groups will be sealed in opaque envelopes, which will be numbered sequentially. The envelopes will not be delivered and opened by a study coordinator until written consent forms and baseline assessments have been completed. The study coordinator will then inform eligible participants of their group allocation and schedule of training sessions by telephone. This trial will be conducted in five cycles of at least 18 participants per cycle for a 12-week intervention course, to ensure a consistent environment for interventions and to maintain an equal number of participants in each group.

### Blinding

Due to the nature of the interventions, instructors and participants will not be blind to group allocations. The study coordinator will be responsible for informing participants of randomized results and scheduling interventions. Data managers, statisticians and evaluators will be blinded to the group allocation throughout the entire procedure to minimize the risk of bias.

### Interventions

#### Tai Chi group

**Fig. 2:**
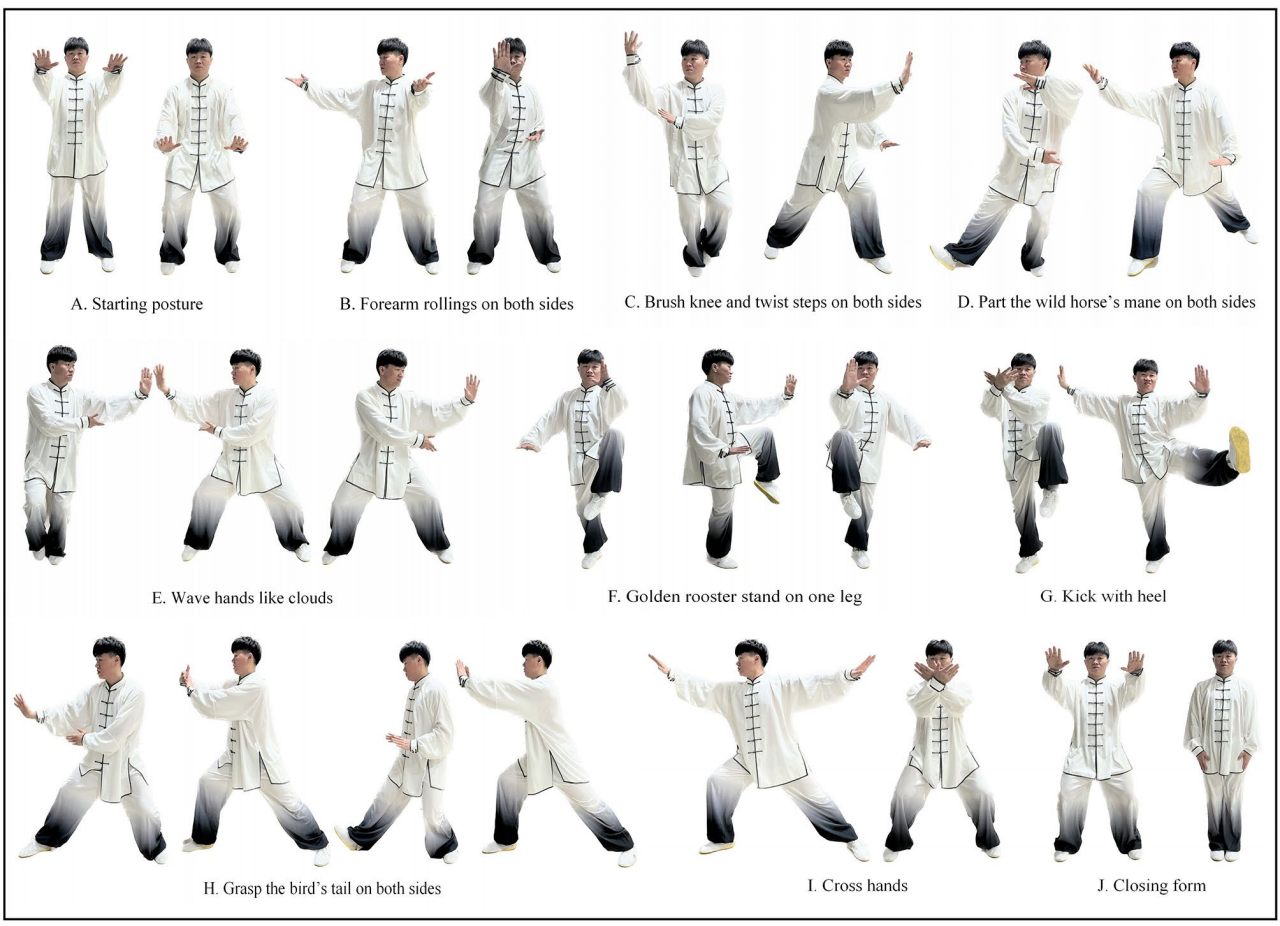
All movements of 8-form Tai Chi

Based on the guidance of Tai Chi masters and previous high-quality KOA studies [25, 29], this simplified Yang-style Tai Chi protocol will be adopted for KOA participants to avoid sustained weight bearing, dynamic rotation of knee joints, and excessive knee flexion. Eight-form Tai Chi is expected to be easily comprehensible for older participants and more suitable for physical conditions of knee osteoarthritis without excessive stress on the joints. Participants in TC group will receive a 60-minute weekly training session for 12 weeks. Each session consists of 10 min of warm-up, 40 min of Tai Chi practice and 10 min of cool-down activities. The training protocol includes eight Tai Chi forms: starting posture, forearm rollings on both sides, brush knee and twist steps on both sides, part the wild horse’s mane on both sides, wave hands like clouds, golden rooster stand on one leg, kick with heel, grasp the bird’s tail on both sides, cross hands and closing form. All movements of 8-form Tai Chi are shown in Fig. 2. The protocol will follow an easy-to-tough pattern, with the first phase (week 0–1) focusing on communicating effectively with participants and practicing Tai Chi preparatory movements, the second phase (week 2–4) focusing on learning and practicing eight Tai Chi forms, and the third phase (week 5–12) focusing on practicing and strengthening the sequence and precision of the forms under the guidance of the instructor.

Participants will also be instructed to practice Tai Chi for 30 min daily at home. Pre-recorded Tai Chi training videos on a WeChat mini-app are made to prevent participants from forgetting details while practicing at home. All participants will be encouraged to maintain their usual physical activities and to perform no additional strength training other than Tai Chi exercises.

#### Attention control group

Participants in the control group will receive a 60-minute weekly health education and stretching program for 12 weeks. It has been successfully applied in other studies as passive control [22, 29], aimed at controlling attention from individuals, motivating enthusiasm for recruitment, attendance, and adherence without affecting main outcomes during the trial. Each session starts with 40 min of lectures on KOA knowledge, including diagnostic criteria, diet and nutrition, pain management, wellness and lifestyle changes, physical and mental health education, and medical management. Orthopedic surgeons, physiatrists, and health consultants from hospitals will deliver these educational lectures for participants. The final 20 min of the hourly session consists of stretching exercises involving the upper body, trunk and lower body. Participants will also be instructed to practice stretching exercises for 30 min daily at home. All participants will be encouraged to maintain their usual physical activities and to perform no additional strength training other than stretching exercises.

### Outcome measurement

#### Primary outcome

The primary outcome is the change in WOMAC pain subscale between baseline and week 12. The self-reported WOMAC pain subscale is aimed to assess pain intensity when walking on a flat surface, going up or down stairs, lying in bed at night, sitting or lying, and standing upright [31]. WOMAC has three subscales evaluating joint pain (score range, 0-500), stiffness (0-200) and physical function (0-1700), respectively, with higher scores indicating more severe disease. The visual analogue scale (VAS) version of WOMAC is a disease-specific, self-administered instrument and widely used in clinical trials with satisfactory reliability and validity [32–34].

#### Secondary outcome

**WOMAC** WOMAC pain subscale assessed at week 6 and 18, stiffness and physical function subscales assessed at week 6, 12 and 18 will be evaluated as secondary outcomes.

#### Measures of muscle strength

Maximal voluntary muscle strength of extensor and flexor will be measured by isokinetic dynamometry at angular velocities of 30 deg/s and 60 deg/s (Biodex System 4 Pro, Biodex Medical System, NY, USA) at baseline and week 12. Before the test, the patient will be comfortably positioned on dynamometry with the hip at 85 deg and the knee at 90 deg flexion. The shin will be placed and strapped in the foot adapter and the rotation axis will be aligned with the flexion and extension axis of the knee. The patient will then be instructed to perform flexion and extension according to his or her physiological capability within the range of motion (ROM) of the knee joint during a 5-minute warm-up period to familiarize himself with the test procedure. A gravitational correction will be applied to negate the influence of the gravity-effect torque on the test data. During the formal test, each patient will be required to fold their arms across their chest and given verbal encouragement in an attempt to achieve a maximum level of effort. The angular velocities of 30 deg/s and 60 deg/s will be applied to two sets of five maximal knee extensions and flexions with a 60-second rest interval between sets, which are chosen according to previous studies [22, 35]. The mean of each set will be selected as maximal isokinetic muscle strength. The peak torque (PT), peak work (PW), total work (TW), peak torque angle (PTA) and average power (AP) of quadriceps were recorded for each velocity.

#### Gait analysis with electromyography (EMG)

At the Biomechanical Laboratory at Yueyang Hospital of Integrated Traditional Chinese and Western Medicine, participants will be instructed to walk along a 12-meter walkway at a comfortable pace, with gait cycles for each limb recorded for 3 trials per established protocol. A 28-reflective marker set, 16-camera Motion Analysis System (Vicon Vero 2.2, Vicon, UK, 100 Hz), 4-channel force plates (AMTI BP400600, AMTI, USA, 1000 Hz), a 6-meter pressure distribution measurement platform (FDM-3, Zebris, Germany) and 10 non-invasive wireless EMG electrodes (Noraxon Ultium, Noraxon, USA) will obtain 3D kinematic and kinetic gait data, which can measure the physical function of the knee joint and neuromuscular function of the lower limbs. Maximal knee compressive force, external knee adduction moment, AP shear force, vertical ground reaction forces, 3D moments and forces of hip, knee and ankle joints will all be analyzed by Vicon motion analysis software (Vicon Nexus 2.12.1, Vicon, UK) and Visual 3D gait analysis software (Visual 3D Standard 4.0, C-Motion, USA). Maximal Voluntary Contraction (MVC) will be collected and analyzed by MyoMuscle EMG analysis software (Noraxon MR3 3.16.20, Noraxon, USA). In addition, gait kinematics of the lower limbs also include total time of one gait cycle (s), velocity (cm/s), step length (cm), knee joint angles (°) during walking.

#### 36-item short form health survey

The 36-item Short Form Health Survey (SF-36) is the most widely used and carefully validated measurement of health related to quality of life [36, 37], consisting of eight dimensions: physical functioning (PF), role limitations due to physical problems (RP), bodily pain (BP), general health (GH), vitality (VT), social functioning (SF), role limitations due to emotional problems (RE), and mental health (MH). Each item is scored on a scale of 0 to 100 with a higher score indicating a better quality of life. In this study, SF-36 will be used to evaluate the effects of Tai Chi on the physical and mental health of KOA patients.

#### Dosage of pain-relieving medication

Participants will be allowed to continue their routine medications to alleviate pain, such as nonsteroidal anti-inflammatory drugs (NSAIDS) and acetaminophen. Any changes in dosage should be recorded in case report form (CRF), but changes to the medical regimen will not be recommended by researchers. The dose dairy is a notepad in which participants will be required to record their daily dose of pain-relieving medication and the duration of the dose.

### Adherence

At the beginning of the study, a group of people interested in Tai Chi will be selected to volunteer for the trial. Participants have the right to choose whether or not to participate after a thorough understanding of the assigned groups and procedures. Participants will be encouraged and informed of the detailed exercise contents and benefits once a week via WeChat or telephone. Researchers will visit training sessions and regularly review course videos to monitor the veracity of interventions. Their attendance at each session will be recorded in attendance forms by study assistants who are not involved in intervention, evaluation and data analysis. We will track the reasons for missed sessions and the number of missed sessions. In addition, participants will be required to upload photos or videos of home exercises to provide feedback to instructors and will be encouraged to complete their daily punches on a WeChat mini-app. Pre-recorded Tai Chi training videos will be made to prevent participants in TC group from forgetting details while practicing Tai Chi at home.

### Safety evaluation

In this study, the categories and severity of adverse events (AEs) should be recorded on the CRF in detail after each intervention, such as falls, joint injuries, hypertension, headache, dizziness, lumbago, tinnitus, chest oppression, etc. Incidence of AEs will be reported as the number of AEs by number of Tai Chi sessions. If AEs do not improve after adequate rest, prompt medical treatment should be applied to the participants. In the event of a severe adverse event (SAE), the principal investigator and ethics committee should be fully informed within 24 h of the occurrence, who are expected to provide guidance for further evaluation and management and make decisions on whether to suspend the trial. Investigators should also pay sustained attention to participants who experience any AE until it is resolved, especially those who have withdrawn due to AEs. In addition, participants will receive appropriate medical and financial compensation if AEs have been shown to be associated with the interventions in this study.

### Quality control

Quality control will be carried out under the supervision of the independent steering committee to eliminate potential bias and ensure the quality of the trial. Steering committee consists of investigators with expertise in Tai Chi clinical trials, statistical design, exercise, and adverse events. They are responsible for monitoring data, identifying problems, reviewing collected data, and controlling bias. To ensure consistency in delivery, the protocol will remain the same throughout the 12-week training period. All instructors and assessors must receive standard training prior to the commencement of the trial. The Tai Chi instructor with extensive experience will be required to receive standard training sessions by a nationally recognized academic specialist with more than 20 years of Tai Chi training prior to the initiation of this trial. Training sessions should be conducted in strict accordance with the Tai Chi protocol, including Tai Chi forms, Tai Chi principles, breathing techniques, concentration, and KOA precautions. Both groups will be conducted simultaneously in small groups with at least 9 participants per class to avoid varying severity of KOA depending on the seasons and weather. For participants, they must upload photos or videos of home exercises to provide feedback to instructors and are encouraged to complete their daily punches on a WeChat mini-app. No additional exercises will be assigned to them during the study period. To maintain consistency in individual Tai Chi intensity, all participants will be required to exercise at a moderate intensity of 50-70% of maximum heart rate (calculated as 220-age, beats/minute) for 60 min. In this study, both heart rate and activity intensity will be recorded using Actigraph wGT3X-BT (ActiGraph LLC, Pensacola, FL), which will be worn on the patient’s waists during Tai Chi at the 1st, 6th and 12th intervention. To ensure pre-specified exercise intensity, participants will be trained to wear Actigraph before exercise to measure intensity. If necessary, the Tai Chi instructor will adjust the number and range of movements to achieve the pre-specified intensity. As for pain-relieving medications, participants will be allowed to continue their routine medications to alleviate pain. Any changes in dosage should be recorded in CRFs, but changes to the medical regimen will not be recommended by researchers.

#### Data collection and statistical analysis

A standardized CRF has already been designed prior to the study, including participants’ characteristics, outcome measures, evaluation time points, adverse events, etc. Two independent data administrators, who are blind to group allocation, will check CRF data and double-enter it into an Excel database once a week. Raw data can be shared by contacting researchers if requested within 6 months of completion of the trial.

A complete analysis set (FAS) is based on the ITT principle, which includes all qualified participants who have received at least one intervention and at least one outcome measurement. Missing data will be imputed through last observation carried forward (LOCF) methodology. A Per-Protocol Set (PPS) will also be used to analyze those who completed the trial without major protocol violations as an auxiliary set. The analysis will be based on the intention-to-treat principle and include all randomly assigned participants receiving interventions. Statistical analysis will be carried out by an independent statistician using SPSS version 26.0. Descriptive characteristics will be presented as mean ± standard deviation or median (interquartile range, IQR) for continuous variables and n (%) for categorical variables. Analysis of variance (ANOVA) or Chi-square test will be used to verify baseline balances of variable characteristics between groups, as appropriate. Changes in repeat outcomes over time will be compared by linear-mixed effects model with group and visits as fixed effects. A Bonferroni correction will be used for multiple comparisons. Statistical significance will be set at a 2-tailed p-value of 0.05.

## Discussion

KOA patients often experience a variety of symptoms that interfere with their daily activities. There are four main symptoms, including pain, stiffness, reduced joint motion and muscle weakness [38], which are interrelated rather than independent of each other. Muscle weakness is a hallmark of KOA patients and a better predictor of disability than pain or narrowing of the joint gap [39]. Decreased muscle strength can reduce knee stabilizers and proprioception, increase the risk of falls and accelerate progression of degenerative KOA [8, 39]. Several studies have found that strengthening the quadriceps is effective in relieving knee pain, reducing joint mobility, and delaying KOA degeneration [9, 11, 12]. Therefore, pain relief is the primary demand to seek KOA treatment. Muscle strength could be the key to treating KOA-associated pain.

TC, as a kind of KOA exercise therapy, is widely considered to be a suitable exercise therapy for KOA patients. Significant improvements have been reported in pain, muscle strength, physical function, balance, mental health and other arthritic symptoms [25, 40]. Some researchers also believe TC activates neuroendocrine and autonomic nervous functions, secretes neurochemicals, triggers an analgesic pathways response, and then regulates inflammatory response of the immune system [41, 42]. The benefits of Tai chi for KOA have been confirmed, but mostly though self-reported symptoms and function outcomes, ignoring muscle strength and the association with pain and physical function. Our primary therapeutic goal is to examine whether Tai Chi can increase quadriceps strength, thereby reducing pain levels, improving physical function and slowing KOA progression.

While the purpose of this trial is to address the limitations of previous trials, potential challenges remain to be addressed. First, to ensure the successful implementation of the protocol, all researchers are trained prior to the start of the trial and are prepared to properly treat patients through systematic research and detailed protocols. Secondly, to increase the representativeness of the sample, public recruitment will be carried out through posters, social platforms and other forms. Third, to improve the accuracy of the exercise, 8-form Tai Chi is adopted for the elderly, which is easy to understand and beneficial for physical conditions without excessive stress on the joints. Participants will be instructed by Tai Chi instructor with extensive experience and reminded of the forms by pre-recorded videos. Fourth, to achieve patient compliance and minimal follow-up loss, We have successful recruitment experience in our previous research with KOA with a low dropout rate. We will use the following strategies. Clinical trial publicity materials are distributed to all researchers to popularize clinical trial knowledge. Before assigning informed consent, participants should be explained in detail what to look for, including intervention requirements, timelines, positive impacts, and potential adverse events. In addition, we will strengthen KOA medical education, care for patients, and improve the overall quality of life of patients. Participants will receive an adequate transportation allowance if they complete all treatment and follow-up. More importantly, more flexible methods will be used to measure patients during follow-up, such as telephone questions or electronic CRF production through information or other communication software.

TC is easy to learn and self-administered and is not limited by time or space. If TC can improve clinical symptoms in KOA patients, we should promote it in the community in the future. The findings will help determine whether TC is superior to regarding effects on knee pain, physical function and muscle weakness in people with KOA and explore its correlation between pain intensity and muscle strength.

### Trial status

At the time of submission, recruitment for the trial has been started. Te first participant was included on 25 April 2023.

## Data Availability

Not applicable.

## Abbreviations

AC: Attention Control
AEs: Adverse Events
ANOVA: Analysis of Variance
AP: Average Power
BP: Bodily Pain
CONSORT: Consolidated Standards of Reporting Trials
CRF: Case Report Form
EMG: Electromyography
GH: General Health
IQR: Interquartile Range
KOA: Knee Osteoarthritis
LOCF: Last Observation Carried Forward
MH: Mental Health
MVC: Maximal Voluntary Contraction
NSAIDS: Nonsteroidal Anti-inflammatory Drugs
PF: Physical Functioning
PPS: Per-Protocol Set
PT: Peak Torque
PTA: Peak Torque Angle
PW: Peak Work
RE: Role Limitations due to Emotional Problems
RP: Role Limitations due to Physical Problems
SAE: Severe Adverse Event
SF: Social Functioning
SF-36: 36-item Short Form Health Survey
SPIRIT: Standard Protocol Items:Recommendations for Interventional Trials
TC: Tai Chi
TESS: Treatment Emergent Symptom Scale
VT: Vitality
TW: Total Work
WOMAC: Western Ontario and McMaster Universities
